# Fast Dissolving Tablets of Fexofenadine HCl by Effervescent Method

**DOI:** 10.4103/0250-474X.54272

**Published:** 2009

**Authors:** D. Nagendrakumar, S. A. Raju, S. B. Shirsand, M. S. Para, M. V. Rampure

**Affiliations:** Department of Pharmaceutics, S. V. E. T. College of Pharmacy, Humnabad-585 330, India; 1 Department of Pharmaceutical Technology, H. K. E. Society's College of Pharmacy, Sedam Road, Gulbarga-585 105, India

**Keywords:** Fexofenadine HCl, fast dissolving tablets, croscarmellose sodium, crospovidone, sodium starch glycolate, effervescent method

## Abstract

In the present work, fast dissolving tablets of fexofenadine HCl were prepared by effervescent method with a view to enhance patient compliance. Three super-disintegrants viz., crospovidone, croscarmellose sodium and sodium starch glycolate along with sodium bicarbonate and anhydrous citric acid in different ratios were used and directly compressible mannitol (Pearlitol SD 200) to enhance mouth feel. The prepared batches of tablets were evaluated for hardness, friability, drug content uniformity and *in vitro* dispersion time. Based on the *in vitro* dispersion time (approximately 20 s), three formulations were tested for *in vitro* drug release pattern in pH 6.8 phosphate buffer, short-term stability at 40°/75% RH for 3 mo and drug-excipient interaction (IR spectroscopy). Among the three promising formulations, the formulation ECP_3_ containing 8% w/w of crospovidone and mixture of 24% w/w sodium bicarbonate 18% w/w of anhydrous citric acid emerged as the best (t_50%_ 4 min) based on the *in vitro* drug release characteristics compared to conventional commercial tablet formulation (t_50%_ 15 min). Short-term stability studies on the formulations indicated that there are no significant changes in drug content and *in vitro* dispersion time (*P*<0.05).

Despite of tremendous advancements in drug delivery, the oral route remains the perfect route for the administration of therapeutic agents because the low cost of therapy and ease of administration lead to high levels of patient compliance. Patient convenience and compliance oriented research has resulted in bringing out safer and newer drug delivery systems. Recently fast dissolving drug delivery systems have started gaining popularity and acceptance as one such example with increased consumer choice, for the reason of rapid disintegration or dissolution, self administration even without water or chewing. Recent advances in technology have presented viable dosage alternatives for patients who have difficulty in swallowing tablets or capsules.

Many patients find it difficult to swallow tablets and hard gelatin capsules and thus do not comply with prescription, which results in high incidence of non-compliance and ineffective therapy. Recent advances in novel drug delivery systems (NDDS) aim to enhance safety and efficacy of drug molecule by formulating a convenient dosage form for administration and to achieve better patient compliance; one such approach is fast dissolving tablets[1–4]. Fexofenadine HCl (FXD), is a non-sedating antihistamine used in the symptomatic relief of allergic conditions including seasonal allergic rhinitis and urticaria[5]. The concept of formulating fast dissolving of tablets containing fexofenadine HCl offers a suitable and practical approach in serving desired objective of faster dissolving and dissolution characteristics with increased bioavailability.

FXD was a gift sample from Aurobindo Pharmaceuticals, Hyderabad, India. Sodium starch glycolate (SSG), croscarmellose sodium (CCS) and crospovidone (CP) were gift samples from Wockhardt Research Centre, Aurangabad, India. Directly compressible mannitol (Pearlitol SD 200) and sodium stearyl fumarate (SSF) were generous gifts from Strides Acrolabs, Bangalore, India. All the other chemicals used were of analytical reagent grade.

For the preparation of fast dissolving tablets, effervescent method[6] was used. All the ingredients (except SSF and purified talc) were accurately weighed and sifted through # 44 mesh separately, sodium bicarbonate and anhydrous citric acid were pre-heated at a temperature of 80° to remove absorbed/residual moisture and were thoroughly mixed in a mortar to get a uniform powder and then added to other ingredients. The ingredients after sifting through sieve No. 44 were thoroughly mixed in a tumbling cylindrical blender (fabricated in our laboratory). The blend thus obtained was directly compressed into tablets of 150 mg weight on a 10-station rotary machine (Clit, Ahmedabad, India) using 8 mm round flat punches. The tablets were prepared according to the formulae shown in Table 1.

**TABLE 1:** COMPOSITION OF DIFFERENT BATCHES OF FAST DISSOLVING TABLETS OF FEXOFENADINE HYDROCHLORIDE

Ingredients* (mg)	Formulation Code#									
	
	EC_0_	ECP_1_	ECP_2_	ECP_3_	ECCS_1_	ECCS_2_	ECCS_3_	ESSG_1_	ESSG_2_	ESSG_3_
Fexofenadine HCl	30.00	30.00	30.00	30.00	30.00	30.00	30.00	30.00	30.00	30.00
Sodium bicarbonate (8-24%)	24.00	12.00	24.00	36.00	12.00	24.00	36.00	12.00	24.00	36.00
Citric acid (6-18%)	18.00	9.00	18.00	27.00	9.00	18.00	27.00	9.00	18.00	27.00
Crospovidone	--	3.00	6.00	12.00	--	--	--	--	--	--
Croscarmellose sodium	--	--	--	--	3.00	6.00	12.00	--	--	--
Sodium starch glycolate	--	--	--	--	--	--	--	3.00	6.00	12.00
Aspartame	7.50	7.50	7.50	7.50	7.50	7.50	7.50	7.50	7.50	7.50
Flavour	1.50	1.50	1.50	1.50	1.50	1.50	1.50	1.50	1.50	1.50
Sodium stearyl fumarate	1.50	1.50	1.50	1.50	1.50	1.50	1.50	1.50	1.50	1.50
Talc	3.00	3.00	3.00	3.00	3.00	3.00	3.00	3.00	3.00	3.00
Pearlitol SD 200	64.5	82.50	58.50	31.50	82.50	58.50	31.50	82.50	58.50	31.50
Total weight	150.0	150.0	150.0	150.0	150.0	150.0	150.0	150.0	150.0	150.0

* Quantity expressed is in mg/tablet.

# A batch of 60 tablets was prepared for each formulation

Twenty tablets were selected at random and weighed individually. The individual weights were compared with the average weight for determination of weight variation[7]. Hardness and friability of the tablets were determined by using Monsanto hardness tester and Roche friabilator, respectively. For determining the content uniformity, ten tablets were weighed and powdered. The powder equivalent to 30 mg of FXD was extracted into methanol and liquid was filtered (Whatman No. 1 filter paper). The FXD content in the filtrate was determined by measuring the absorbance at 259 nm after appropriate dilution with methanol. The drug content was determined using the standard calibration curve. The mean percent drug content was calculated as an average of three determinations[8]. For determination of *in vitro* dispersion time, one tablet was placed in a beaker containing 10 ml of pH 6.8 phosphate buffer at 37±0.5° and the time required for complete dispersion was determined[9]. IR spectra of FXD and its formulations were obtained by potassium bromide pellet method using Perkin-Elmer FTIR series (Model 1615) spectrophotometer in order to rule out drug-carrier interactions.

*In vitro* dissolution of FXD fast dissolving tablets was studied in USP XXIII type-2 dissolution apparatus (Electrolab, Model-TDT 06N) employing a paddle stirrer at 50 rpm using 900 ml of pH 6.8 phosphate buffer at 37±0.5° as dissolution medium. One tablet was used in each test. Aliquots of dissolution medium (5 ml) were withdrawn at specific intervals of time and analyzed for drug content by measuring the absorbance at 259 nm. The volume withdrawn at each time interval was replaced with fresh quantity of dissolution medium. Cumulative percent of FXD released was calculated and plotted against time.

Short-term stability studies on the promising formulations (ECP_3_, ECCS_3_ and ESSG_3_) were carried out by storing the tablets in amber coloured glass vial with rubber stopper at 40°/75% RH over a 3 mo period (as per ICH guidelines). At an interval of 1 mo, the tablets were visually examined for any physical changes, changes in drug content and *in vitro* dispersion time.

Fast dissolving tablets of FXD were prepared by effervescent method, as this method has the advantage of taste masking. Further, aspartame 5% and 1% flavour was used in the formulations to enhance the mouth feel, employing CP, CCS and SSG as super-disintegrants along with mixtures of sodium bicarbonate and anhydrous citric acid in different ratios. Directly compressible mannitol (Pearlitol SD 200) was used as a diluent to enhance mouth feel[9]. A total of nine formulations and a control formulation EC_0_ (without super-disintegrant) were designed[10]. As the blends were free flowing (angle of repose <30°, and Carr's index <15%) tablets obtained were of uniform weight (due to uniform die fill), with acceptable variation as per IP specifications i.e., below 7.5%. Drug content was found to be in the range of 95 to 101%, which is within acceptable limits. Hardness of the tablets was found to be 2.5 to 2.8 kg/cm^2^. Friability below 1% was an indication of good mechanical resistance of the tablets. Among all the designed formulations, three formulations, viz., ECP_3_, ECCS_3_ and ESSG_3_ were found to be promising and displayed an *in vitro* dispersion time ranging from 19 to 26 s, which facilitates their faster dispersion in the mouth.

Overall, the formulation ECP_3_ containing 8% w/w of crospovidone along with mixture of sodium bicarbonate 24% w/w and anhydrous citric acid 18% w/w was found to be promising and has shown an *in vitro* dispersion time of 20 s when compared to control formulation (EC_0_) which shows 498 s value for *in vitro* dispersion (Table 2).

**TABLE 2:** EVALUATION OF FAST DISSOLVING TABLETS

Parameters	Formulation Code									
	
	EC_0_	ECP_1_	ECP_2_	ECP_3_	ECCS_1_	ECCS_2_	ECCS_3_	ESSG_1_	ESSG_2_	ESSG_3_
Hardness* (kg/cm^2^)±SD	2.53±0.23	2.76±0.25	2.50±0.50	2.63±0.05	2.51±0.14	2.63±0.15	2.60±0.20	2.56±0.15	2.76±0.25	2.63±0.05
Friability(%)	0.60	0.58	0.56	0.54	0.64	0.60	0.52	0.70	0.60	0.52
Thickness (mm)	2.72	2.95	2.93	2.70	2.73	2.78	2.74	2.80	2.92	2.82
*In vitro* dispersion time* (sec)±SD	497.8±4.20	55.8±2.9	38.18±2.00	19.78±1.16	60.21±0.78	40.00±1.50	22.10±0.85	62.00±1.0	43.00±1.00	26.6±1.32
% drug content*±SD	99.45±0.70	99.4±1.0	100.5±0.74	101.3±0.74	95.68±0.59	97.96±1.38	97.76±0.73	97.74±0.62	99.03±0.78	97.74±0.62
Weight variation	(147 – 155 mg) within the IP limits of±7.5%									

* Average of three determinations. Formulations ECP_3_, ECCS_3_ and ESSG_3_ were selected as the best formulations and used for further studies.

*In vitro* dissolution studies on the promising formulations (ECP_3_, ECCS_3_ and ESSG_3_), the control (EC_0_) and commercial conventional formulations (CCF) were carried out in pH 6.8 phosphate buffer, and the various dissolution parameter values viz., percent drug dissolved in 5 min, 10 min and 15 min (D_5_, D_10_ and D_15_), dissolution efficiency at 10 min (DE_10 min_)[11], t_50%_, t_70%_ and t_90%_, the dissolution profiles depicted in fig. 1. This data reveals that overall, the formulation ECP_3_ has shown nearly four-fold faster drug release (t_50%_ 4 min) when compared to the commercial conventional tablet formulations of FXD (t_50%_ 15 min) and released 5-times more drug than the control formulation in 10 min.

**Fig. 1:**
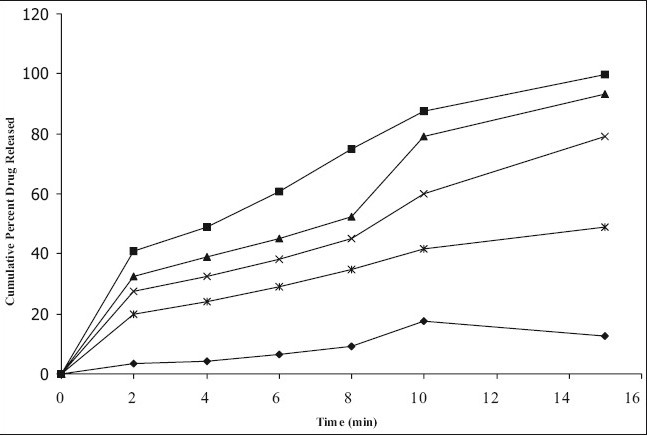
Cumulative percent drug release versus time profile of promising formulations. *In vitro* cumulative percent drug release versus time profile of promising formulations in pH 6.8 phosphate buffer. The prochlorperazine maleate formulations tested were, EC0 (–◆–), ECP_3_ (–■–), ECCS_3_ (–Δ–), ESSG_3_ (–x–), CCF (–*–)

IR spectroscopic studies indicated that the drug is compatible with all the excipients. The IR spectrum of ECP_3_ showed all the characteristic peaks of FXD pure drug, thus confirming that no interaction of drug occurred with the components of the formulation. Short-term stability studies of the above formulations indicated that there are no significant changes in drug content and *in vitro* dispersion time at the end of 3 mo period (*P*<0.05).
